# PRECOG: PREdicting COupling probabilities of G-protein coupled receptors

**DOI:** 10.1093/nar/gkz392

**Published:** 2019-05-30

**Authors:** Gurdeep Singh, Asuka Inoue, J Silvio Gutkind, Robert B Russell, Francesco Raimondi

**Affiliations:** 1 CellNetworks, Bioquant, Heidelberg University, Im Neuenheimer Feld 267, 69120 Heidelberg, Germany; 2 Biochemie Zentrum Heidelberg (BZH), Heidelberg University, Im Neuenheimer Feld 328, 69120 Heidelberg, Germany; 3 Graduate School of Pharmaceutical Sciences, Tohoku University, Sendai, Miyagi 980-8578, Japan; 4 Department of Pharmacology and Moores Cancer Center, University of California, San Diego, La Jolla, CA 92093, USA

## Abstract

G-protein coupled receptors (GPCRs) control multiple physiological states by transducing a multitude of extracellular stimuli into the cell via coupling to intra-cellular heterotrimeric G-proteins. Deciphering which G-proteins couple to each of the hundreds of GPCRs present in a typical eukaryotic organism is therefore critical to understand signalling. Here, we present PRECOG (precog.russelllab.org): a web-server for predicting GPCR coupling, which allows users to: (i) predict coupling probabilities for GPCRs to individual G-proteins instead of subfamilies; (ii) visually inspect the protein sequence and structural features that are responsible for a particular coupling; (iii) suggest mutations to rationally design artificial GPCRs with new coupling properties based on predetermined coupling features.

## INTRODUCTION

G-protein coupled receptors (GPCRs) are the largest class of cell-surface receptors and the target for 30% of marketed drugs (1,2). They are responsible for transducing a myriad of stimuli from the extracellular environment to activate multiple intracellular signalling pathways. They do so by coupling to one or more heterotrimeric G-proteins, whose α-subunits are grouped into four major G-protein families: G_s_, G_i/o_, G_q/11_ and G_12/13_ (3). Aberrant coupling of GPCRs to G-proteins has been linked to several pathological processes and diseases such as cardiovascular and mental disorders, retinal degeneration, AIDS and cancer (4). Untangling GPCR/G-protein coupling can also aid the design of chemogenetic tools, such as Designer Receptors Exclusively Activated by Designer Drugs (DREADDs), that can be of great use in tinkering with signalling pathways in living systems (5).

Ligand binding to GPCRs induces conformational changes that lead to binding and activation of G-proteins situated on the inner cell membrane. Most of mammalian GPCRs couple with more than one G-protein giving each receptor a distinct coupling profile (6) and thus specific downstream cellular responses. Determining these coupling profiles is critical to understand GPCR biology and pharmacology. Despite decades of research and hundreds of observed interactions, coupling information is still missing for many receptors and sequence determinants of coupling-specificity are still largely unknown. However, it is clear that, in contrast to e.g. enzyme specificities (7), simple amino acid differences explaining coupling differences are rare.

Here, we present a machine learning-based predictor (PRECOG) of Class A GPCR/G-protein couplings, which was developed as a part of the most systematic quantification of GPCR coupling selectivity to date (8). PRECOG was built by exploiting experimental binding affinities of 144 human Class A GPCRs for 11 chimeric G-proteins obtained through the TGFα shedding assay (9). We derived a set of sequence- and structure-based features that were statistically associated with each of 11 G-proteins, which we used to devise predictive models.

Given one or more input sequences or Uniprot protein accessions (or gene symbols), PRECOG provides both overview predictions for each G-protein and putative mechanistic insights into how each prediction was made. Determinants of coupling-specificity are displayed on the sequence and on available (known or homologous) 3D structures. Users can also assess the impact of mutations on GPCR/G-protein coupling with respect to the wild type. We provide views that can aid users in selecting mutations that can help alter coupling specificity and ultimately to design receptors having specific couplings. We have already used PRECOG to predict coupling preferences of all human GPCRs, as well as to design a chemogenetic tool (DREADD) specific for *GNA12* (8).

## MATERIALS AND METHODS

### Coupling data for 144 class A GPCRs and 11 chimeric G-proteins from the TGFα shedding assay

To train a predictor for G-protein coupling specificity, we exploited data from the TGFα shedding assay, which is a robust, high-throughput means to measure accumulated GPCR signals (8,9). This approach exploits a ADAM17-induced ectodomain shedding of alkaline phosphatase-fused TGFα (AP-TGFα) and chimeric G-proteins where the 11 unique C-termini (which have previously been shown to account for most of the coupling specificity) from human Gα subunits replace the last 6 amino acids of *GNAQ*. Chimeric G-proteins are expressed in cells lacking endogenous Gα subunits (*GNAQ, GNA11, GNA12* and *GNA13*) that mediate the AP-TGFα shedding response. This means that induction of specific GPCRs with titrated concentration of their ligands leads to binding to the co-transfected G-protein partner. AP-TGFα release signals over titrated concentrations were fitted with a sigmoidal concentration-response curve, from which we obtained EC_50_ and E_max_ values. For each chimeric Gα condition, an E_max_/EC_50_ value was normalized by the maximum E_max_/EC_50_ value among the 11 Gα chimeras (relative intrinsic activity, RAi (10)). The base-10 log-transformed values (LogRAi), ranging from –2 to 0 (100-fold in linear range), represent coupling indices. We have shown that the chimeric G-proteins, with their C-termini, are capable of reporting a reliable coupling across the four G-protein families (8). Functional assays were performed systematically for 144 representative Class A GPCRs. In order to define a LogRAi threshold for true couplings, we compared our dataset with reported couplings from the IUPHAR/BPS Guide to PHARMACOLOGY (GtoPdb) (6) through a Receiver Operating Characteristic (ROC) analysis, which suggested a cutoff of LogRAi ≥ –1.0 (optimizing True Positive Rate, or TPR, while minimizing False Positive Rate, or FPR; AUC = 0.78) when considering high-confidence known coupling data (8).

The use of individual genes instead of the standard coupling groups confuses nomenclature. For clarity, we use group symbols (G_q/11_, G_i/o_, G_s_, G_12/13_) when speaking of the collective action of all proteins in each group, and gene symbols when referring to specific proteins. The 11 subunits grouped are: G_q/11_ = *GNAQ, GNA14, GNA15*; G_s_ = *GNAS, GNAL*; G_12/13_ = *GNA12, GNA13*; G_i/o_ = *GNAI1, GNAI3, GNAO1, GNAZ*. We note that the six C-terminal sequences are identical for *GNAQ* and *GNA11*, and for *GNAI1, GNAI2, GNAT1, GNAT2* and *GNAT3* and that these members are not distinguished in our analyses.

### Feature generation

We constructed a multiple sequence alignment of the 144 Class A GPCR sequences through the HMMalign tool from the HMMER3 package (version 3.1b2 (February 2015)) (11) (see Supplementary Dataset 1), using the 7tm_1 Hidden Markov Model (HMM) from Pfam (2016 release) (12). We then subdivided sequences into positives (coupled; LogRAi ≥ –1) and negatives (not-coupled; LogRAi <-1) for each G-protein. We then extracted sub-alignments and constructed their corresponding HMM profiles (coupled vs. not-coupled for 11 G-proteins) using HMMbuild (11).

For a given G-protein, we then extracted positions showing statistically significant differences in terms of the amino acid bit-scores (Wilcoxon's signed-rank test; *P*-value ≤ 0.05) among the coupled and uncoupled HMMs. Alignment positions with consensus columns (i.e. having a fraction of non-gaps equal or greater than the *symfrac* parameter, considering a default value of 0.5) present in either HMMs, were considered as either insertion or deletion if they were present only in the coupled or not-coupled group. We also included length and amino acid composition of the third intracellular loop (ICL3) and C-terminus (C-term) considering features showing statistically significant differences (*P*-value < 0.05; Wilcoxon's rank-sum test) in coupled vs not-coupled.

We employed the Ballesteros/Weinstein (B/W) scheme (13) to number alignment positions (using GPCRDB (14) to define the most conserved position). For positions lying outside of the transmembrane helices (e.g. ICL3), we note the corresponding Pfam 7tm_1 position in parenthesis.

We integrated the above sequence-based feature set with additional structure-based features derived from available 3D complex structures of Class A GPCRs/G-proteins through the InterPreTS approach (15,16), which uses learned parameters of amino-acid pair contacts across protein interfaces (i.e. statistical potentials) to predict how well aligned homologues fit on to a particular interface of known structure. We selected six GPCR-G protein complex structures covering the most diverse interaction interface repertoire (considering both receptors and G-proteins): *ADRB2-GNAS* (PDB ID: 3SN6), *ADORA2A*-mini*GNAS* (6GDG), *RHO-GNAI1* (6CMO), *Oprm1-GNAI1* (6DDE), *ADORA1-GNAI2* (6D9H), *HTR1B-GNAO1* (6G79). For each complex, we aligned GPCR and chimeric Gα subunit sequences from the TGFα shedding assay to sequences homologous to the corresponding template structure chains. For each template structure, we calculated Z-scores and *P*-values (by generating 100 random permutations) for all the 144 Class A GPCR with each of the 11 G-proteins generating score distributions for coupled and uncoupled receptors to a particular G-protein and checking, through a Wilcoxon rank-sums test (*P* < 0.05), whether these were significantly different among the two groups. Whenever true, we considered that 3D complex as suitable to model the interaction with a particular G-protein and included derived *Z*-scores as features in the model.

### Predictor

We implemented the predictor using a logistic regression (log-reg) classifier, available from the Scikit-learn package (17), considering the features described above. A logistic regression model is defined as:

(1) \begin{equation*}{h}({x}) = {w}_1{x}_1 + {w}_2{x}_2 + {w}_3{x}_3 + \ldots . + {w_nx_n}\end{equation*}

where *x*_1_, *x*_2_, *x*_3_ …, *x*_*n*_ are input features whereas *w*_1_, *w*_2_, *w*_3_, …, *w*_*n*_ denote the regression coefficients. Thus, the probability of the input to couple with a given G-protein can be defined as:

(2) \begin{equation*}f(x) = {(1 + {e^{ - {W^{T}} \,X}})^{ - 1}}\end{equation*}

where the variable *X* and *W* denote the vector of input features [*x*_1_, *x*_2_, *x*_3_, …, *x*_*n*_] and of the regression coefficients [*w*_1_, *w*_2_, *w*_3_, …, *w*_*n*_], also termed *weights*, respectively.

Regularization is an essential technique in machine learning to counter over-fitting, which log-reg implements in two forms: L1 and L2. Both have a Lambda parameter that is directly proportional to the penalty of finding complex or over-fitted models. The regularization term (i) in the L1 form is the product of Lambda and the sum of the weights, while (ii) in the L2 form (used here) it is the product of Lambda and the sum of the squares of the weights. The target value is expected to be a linear combination of the features considered.

As an optimization problem, binary class L2 penalized logistic regression minimizes the following cost function:

(3) \begin{equation*}\mathop {\min }\limits_{w,c} \left(\frac{1}{2}{w^T}w + C\mathop \sum \limits_{i\ = \ 1}^n {\rm{log}}({\rm{exp}}( - {y_i}(X_i^Tw + c)) + 1)\right)\end{equation*}

where *c* ∈ R∧n is the intercept, *C* is inverse of regularization strength (positive float), *y* takes values in {–1, 1} at trial *i* and *n* is the number of trials conducted. We used the *liblinear* method as the optimization algorithm as shown to be optimal for relatively small datasets (18).

Considering 7TM positional, extra-domain and structural features, we created a training matrix for each G-protein. For positional features, every position in the input sequence provided two bit scores (derived from the coupling and not-coupling HMMs for a given G-protein) for the corresponding residue. For insertions or deletions, the approach returns the single bit score, derived from the respective HMM (i.e. coupled or not-coupled). If for any GPCR, no amino acid was present at the given position, it is assigned the highest bit scores from both the models, implying the least conserved scores.

We scaled all the features in the training matrix to the range [0, 1], which helps both to converge the algorithm faster and to assess the feature relevance (19). We performed a subsequent grid search over a stratified 5-fold cross validation to select the best value of *C* (inverse of the regularization strength). Owing to the imbalance nature of the set, we set the *class_weight* parameter to *balanced*, which automatically adjusts the weights of the classes (coupled versus not coupled) inversely proportional to their frequencies in the training matrix. We divided the training matrix randomly into five equally stratified sub-matrices, preserving the ratio of positive (coupled) and negative (not coupled) GPCRs. To build a model for each G-protein, we chose the parameters showing the best Area Under the Curve (AUC) of the ROC curve. We repeated the experiments ten times, for each G-protein, to ensure minimal variance due to random division of the training matrix during cross validation. We assessed the performance of our predictor using standard metrics (MCC, ACC, PRE, REC, SPE, AUC, F1M; Supplementary Table S1). We used the weights obtained after the training of the logistic regression model (as in (19)) to highlight the most relevant features of every G-protein group, which can also be seen as a heat-map (see Supplementary Figure S1).

We performed a randomization test to assess over-fitting (20), where we replace the original G-protein labels of the training matrix with randomly assigned labels, while preserving the ratio of number of positive (coupled) and negative (not coupled) GPCRs (Supplementary Table S2).

### Pipeline

**Figure 1.**
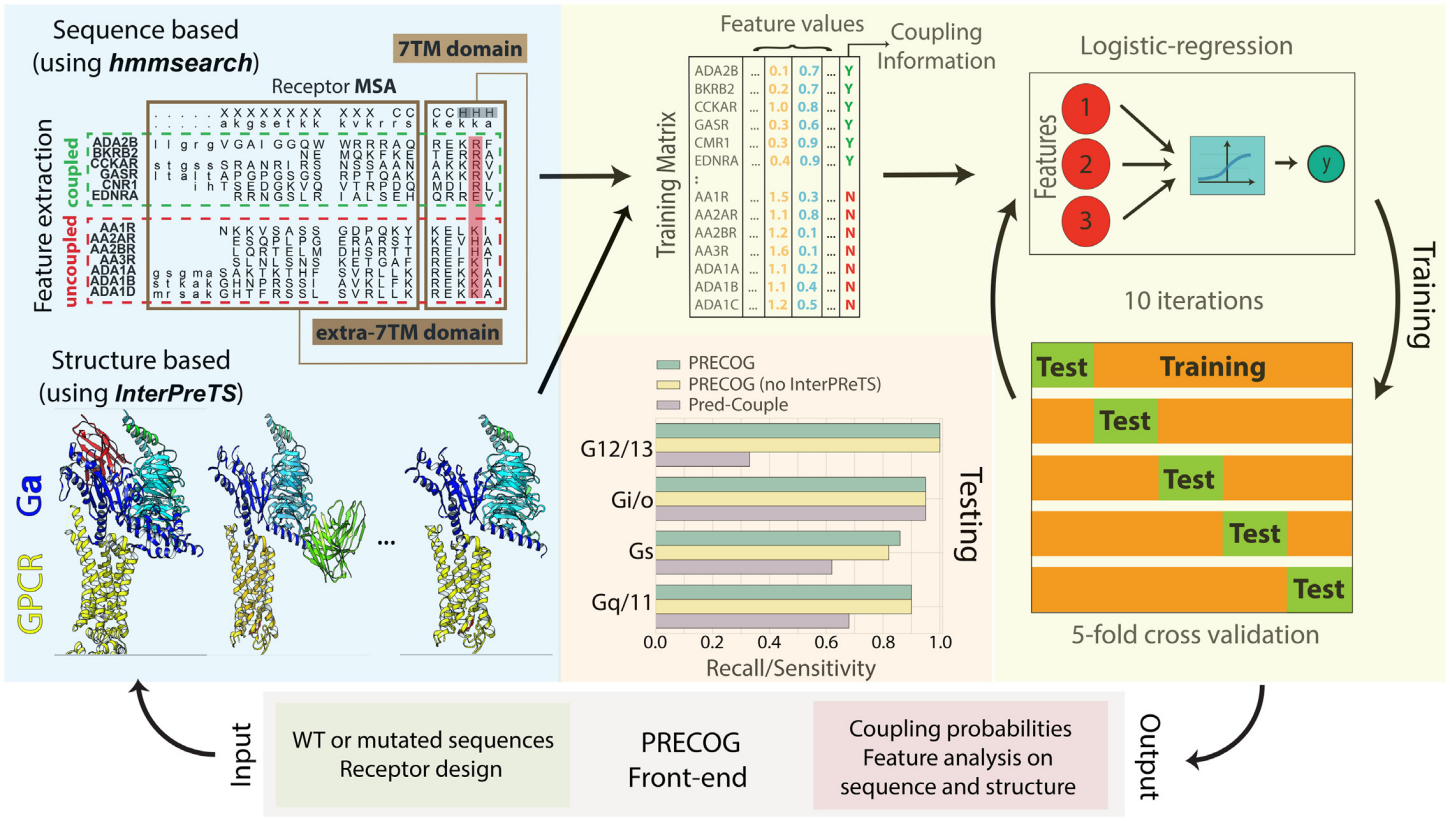
Workflow of the procedure. The user queries the server by inputting either the receptor sequence or mutations through the front-end. Features are extracted from the sequences and used by the machine learning algorithm to carry out the predictions. Results are returned to the front-end and summarized in a tabular format as well as annotated into sequence and structural representations for in depth analysis.

Given user input data, i.e. receptor WT or mutant sequences, the web server internally performs the following key steps to extract features (see Figure 1). First, the input sequences are aligned through *hmmsearch* to the 7tm_1 HMM model to get the sequence aligned to the 7TM helices and to be assigned the consensus B/W numbering (see above). From the coupled and not coupled HMMs of each of the 11 G-proteins, bit-scores of the corresponding amino acid at relevant positions are extracted and insertions/deletions are detected and used as features for predictions. Additional features are obtained by calculating the length and amino acid compositions (e.g. ICL3 and C-terminus).

Second, InterPreTS is run with default parameters, performing 100 random permutations, to derive scores (for each individual input sequence) that predict the plausibility of interaction between the input and chimeric G-protein sequences according to available 3D complex structures (see above). For each 3D complex, InterPreTS takes the corresponding structure and multiple sequence alignments of receptors and G-proteins.

Additionally, to detect the closest homolog for structural visualization purposes, every input sequence is aligned through BLAST (21) to 3D structures of Class A GPCRs from the PDB (nearly 250 structures to date), obtained from SIFT PDB-PFAM mappings (22).

We developed PRECOG by using the Python programming language, both for the web framework, which is based on Flask (http://flask.pocoo.org/), and the internal pipeline to handle back-end processes. Additionally, we used several JavaScript libraries at the front end. In more details, we used JSmol (http://www.jmol.org/) to view protein structure in 3D and neXtProt (23) sequence viewer to draw protein sequences in a readable format.

## USING THE WEBSERVER

### Input

The input can be one or more protein identifiers (UniProt identifiers, accessions or gene symbols), mutations or FASTA sequences. A user can choose to make predictions or design a GPCR. The first option allows to predict the coupling preferences for input receptor(s), either wild type or mutant (see Figure 1).

The second option exploits feature information (i.e. weights) to automatically suggest a ranked list of mutations that are more likely to favour (or disfavour) particular couplings. Checkboxes are provided to enable the users to select the members of one or more G-protein families as target of the design. Residues of the input sequence corresponding to 7TM positions that are statistically associated to a coupling(s) of choice are systematically mutated into each of the remaining 19 amino acids and coupling probabilities computed. For each mutant and each G-protein coupling, two probability differences are calculated: *P*(coupled), i.e. the difference between Mutant and WT, and *P*(uncoupled), i.e. the difference between the WT and the Mutant. While the former tells if a particular mutation is predicted to increase a coupling of interest, while the latter suggests whether the same modification reduces unwanted couplings. To shortlist more interesting mutations, it is possible to set a threshold for both probability differences, which is by default 0.25, corresponding to the value that retains most of the interesting candidates based on our experience.

### Output

**Figure 2.**
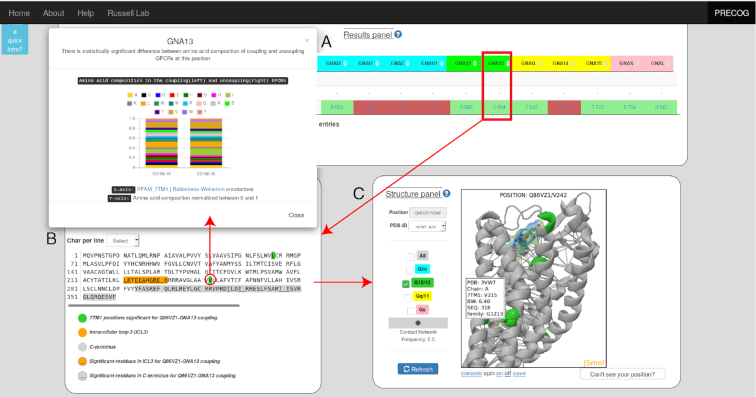
Illustrative example of a prediction to uncover couplings of a poorly characterized receptor (i.e. *P2RY8*). (**A**) Summary table with predicted couplings. Those with coupling probabilities greater than 0.5 are highlighted in green, the others in red. Above each prediction (indicated as P(WT)), couplings from GtoPdb (where PC and SC stand for Primary and Secondary Couplings, respectively) and from the TGFα shedding assay (a LogRAi value equal or greater than –1 indicating coupling); (**B**) query receptor sequence, with highlighted coupling features for a coupling of interest (i.e. *GNA13* in this example). If the length of either ICL3 or C-term is relevant for the prediction, the entire corresponding amino acid stretch is highlighted in orange or grey in the sequence. ICL3 and C-term amino acids whose count is relevant for a given coupling are underscored. Significant 7TM positions are highlighted in the family with specific color code (i.e. green for G_12/13_) and by clicking on each of them a barplot with bitscores distribution from coupled and not-coupled HMMs for that G-protein is displayed. Clicked 7TM significant positions are automatically displayed as spheres on the corresponding position of the closest (by homology) template 3D structure; (**C**) 3D cartoon representation of the closest structure (i.e. 3WL7 for P2RY8, by homology) with positions corresponding to significant features highlighted with the same family-specific color coding (i.e. green for G_12/13_) and links indicating consensus contact network (contact frequency ≥ 0.5).

For both options the user can visualize a summary of predicted couplings as well as the sequence and structural features responsible for predictions (Figures 1 and 2). Figure 2 shows an illustrative example of the output. We have chosen *P2RY8*, a purinergic receptor which has been reported to be recurrently mutated in lymphomas, where it also displays mutual exclusivity with *GNA13* (24–26). Despite these mutations have been functionally linked in cancer, direct experimental evidence of binding is missing and *P2RY8* transduction mechanisms are currently not reported in GtoPdb (6). PRECOG readily predicts *P2RY8* to be a *GNA13* coupled receptor (Figure 2) as is widely expected owing to the observations above.

Users are presented first with an overview showing coupling probabilities of individual G-proteins for each protein or mutant queried (Figure 2A). In addition, when available, information on known couplings (either from GtoPdb or our TGFα shedding assay results) are shown for comparison, including the measured parameters when available (i.e. LogRAi (8)). Moreover, an interactive sequence and structure viewer (Figure 2B,C) shows positions in the sequence and structure that PRECOG identifies as most relevant for any selected G-protein. As for *P2RY8* prediction, projection of feature weights on the sequence as well as on the closest 3D homolog structure, suggests that the strongest contributions to this prediction derive from amino acids at the ICL3 and several 7TM positions (e.g. 6.40; Figure 2B, C).

Interestingly, positions identified as relevant to coupling predictions are not always at known GPCR/G-protein interfaces. We have recently shown that determinants of coupling specificity span the entire 7TM bundle and connect, in a G-protein-specific fashion, the intracellular face with the ligand binding sites through a network of intramolecular residue contacts (8). Additionally, recent studies have emphasized the role of amino acids near or at the ligand binding pocket as triggers for biased agonism (27,28). To highlight these mechanisms, which might be of great relevance in the design of biased ligands, we give the user the opportunity to visualize a consensus network (where links are contacts mediated by 7TM positions, i.e. network nodes), derived from the analysis of multiple 3D structures in both active and inactive states. We moreover show, whenever present in the chosen 3D structure, the ligand (as sticks) and G-protein (as cartoons).

The user is given the option to choose alternative structure templates corresponding to the input sequence through a dropdown menu (by default the closest match by sequence homology is shown, see Methods). With the help of checkboxes, the user can also toggle between the significant positions of G-protein families to be displayed on the structure. Information about interaction contacts, either involving ligand or G-protein binding interfaces, or the network of intramolecular contacts, is obtained by our previous study (8) and can also be optionally visualized on the structure. A widget allows the user to visualize edges at different contact frequency cutoffs. The frequency is calculated as the fraction of protein sequences with at least one structure forming a given contact (8).

## RESULTS

### Test set

We compared the performance of PRECOG with that of PredCouple, a publicly available GPCR/G-protein prediction tool (29) by running both on a list of 86 Class A GPCRs whose coupling is reported in GtoPdb, but which were absent from both training sets (see Supplementary Dataset 2). Since both GtoPdb and PredCouple only consider G-protein families and not specific G-proteins, we grouped PRECOG predictions to this level, considering any G-protein to represent its family. In the absence of any available true negative set, thus, we chose recall (sensitivity or true positive rate) as the metric to compare performances. We also trained and tested an additional predictor using exactly the same procedure as reported above using GtoPdb coupling information instead of the TGFα shedding assay. This allowed us to assess whether our approach, in the absence of a rich new dataset, showed improvement over earlier methods.

Indeed, our finally selected models outperformed both this last predictor as well as PredCouple, indicating the critical contribution from the TGFα shedding assay couplings (Supplementary Table S3).

### Expanding the knowledge of coupling mechanisms of wild type and mutant receptors

The TGFα shedding assay has provided new, quantitative coupling information for well characterized receptors such as *GNAI1/GNAI2* and *GNAZ* for *CHRM3*, and the predictor we have developed proved successful in reproducing them. PRECOG can also be used to illuminate the coupling mechanisms of poorly characterized receptors. For example, for the 61 receptors (21% of 286 Class A GPCRs) lacking coupling information from either GtoPdb or the chimeric G-protein-based assay, we predict a prevalence of G_s_ followed by G_q/11_ and G_12/13_ couplings, the latter being the smallest fraction among currently known experimental couplings (Supplementary Figure S3).

PRECOG can also be used to predict the effect of mutations on G-protein coupling. Many mutations that have been reported to affect GPCR function and couplings (reviewed in (30)), and many have also been annotated in Uniprot to affect signaling (Supplementary Table S4). We systematically investigated the effects of these mutations on coupling through PRECOG, revealing that 68% are predicted to affect coupling (i.e. absolute value of *P*(MUT) – P(WT) ≥ 0.1). The most affected couplings are those of G_s_, G_i/o_ and, to a lesser extent, G_12/13_ (see Supplementary Table S4).

### Adding 3D complex information

We integrated in PRECOG structural information from the increasingly available GPCR/G-protein complexes (31–37). To assess the fit of each 3D complex to model the interaction pairs from the TGFα shedding assay, we used InterPreTS, an approach previously employed for structural annotation of protein interactions (38) (see Methods). As expected, we observed that G_s_ complexes (i.e. PDB IDs: 3SN6 and 6GDG) are statistically associated to the corresponding couplings in the TGFα shedding assay (i.e. *GNAS* and *GNAL*), as well as the *GNAO1-HTR1B* (PDB ID: 6G79) complex is relevant for G_i/o_ couplings (i.e. *GNAI1/GNAI2*) (see Supplementary Table S5). Surprisingly, we found that two more G_i/o_ complexes (i.e. 6CMO and 6DDE) are also good templates to model G_12/13_ couplings, suggesting for this receptor class an interaction topology similar to the G_i/o_ family members (see Supplementary Table S5).

Integration of structure-derived features from 3D complex analysis leads to modest improvement of predictor performance only for the G_s_ family (Supplementary Figure S2). It is likely that additional structures (e.g. including those groups lacking complexes entirely like G_q/11_ or G_12/13_) will lead to additional improvements in the future.

## DISCUSSION

PRECOG represents a significant improvement over previous methods (29,39) both in terms of performance, but also, by way of the web interface, in the ability to interrogate predictions for putative mechanistic explanations that can be used potentially to alter coupling or design receptors *de novo* for particular signalling effects.

The framework that we have developed lends itself naturally to several future enhancements. First, the availability of new data will enable new types of predictions (e.g. other classes of GPCRs and potentially other interactions such as β-arrestin). Easy visualization of coupling determinants on sequence and structure, integrated with the usage of contact networks, that are increasingly employed to understand signalling protein mechanisms (40–49), will ease the rational design of new biased ligands. Second, the speed of the predictions will allow for more ambitious automated design strategies, such as the ability to swap longer, variable segments from multiple GPCRs, as we have employed successfully in the development of a *GNA12* specific DREADD (8). Lastly, this framework can be adopted in the context of any protein-interaction where specificity is difficult to determine from sequence, but for which binding data are available.

## Supplementary Material

gkz392_Supplemental_Files
