# Supplementary material for: PRECOG: PREdicting COupling probabilities of G-protein coupled receptors
Source: Nucleic Acids Res. 2019 May 30;47(W1):W395–401. doi: 10.1093/nar/gkz392 (PMC6602504; doi:10.1093/nar/gkz392)
Supplement: gkz392_Supplemental_Files [file gkz392_supplemental_files.zip › Supplementary Figures S1-S3.pdf]

Figure S1: Heatmap showing features found to be statistically associated (P <0.05) to at least one coupling group (Y axis) for each of the 11 G-protein couplings (X axis). Cell colours are based on coefficients of the given feature for the corresponding coupling group in the decision function of the corresponding coupling group, based on a red-green colour ramp for negative and positive weights. For significant 7TM domain positions characterized by consensus columns in both coupled and uncoupled HMMs, the corresponding feature weights are displayed on the same row.

Figure S2: Recalls for the list of 86 Class A GPCRs, not used to train PRECOG or PredCouple, predicted by PRECOG with or without InterPreTS scores (light green and yellow, respectively) or with PredCouple (light purple).

Figure S3: Highly confident predicted couplings (coupling probability >0.9) for 61 Class A GPCRs lacking information about transduction from both GtoPdb or the chimeric G-protein-based TGFα shedding assay (black) vs. receptors with experimental coupling information (grey).

Table S1: Cross-validation and Test results of the best performing predictors of individual G-proteins on the actual data set at LogRAi >= -1.0.

Table S2: Cross-validation and Test results of the best performing predictors of individual G-proteins on the Randomized set at LogRAi >= -1.0

Table S3: Test results (Recall/Sensitivity) of the best performing predictors at the G-protein family level.

Table S4: Coupling probability differences (P(Mutant) - P(Wild Type)) for Class A GPCR mutations reported in Uniprot to affect signaling.

Table S5: Statistical associations between InterPreTS scores derived from 3D GPCR/G-protein complexes and TGFα shedding assay couplings.

Supplementary Dataset1: Multiple sequence alignment of 144 Class A GPCRs from the chimeric G-protein-based TGFα shedding assay used to train the coupling predictor model. Multiple sequence alignments have been obtained by aligning corresponding input sequences through their 7tm_1 hmm model from PFAM (11) using HMMalign (10). MSA image was generated through Jalview (49) coloring residues according to % identity.

Supplementary Dataset2: Multiple sequence alignment of 86 Class A GPCRs from GtoPdb not in the PRECOG or PredCouple datasets, but with available primary or secondary transduction mechanisms, which we used to test the predictor model. Alignment generation and visualization as above for Supplementary Dataset1.

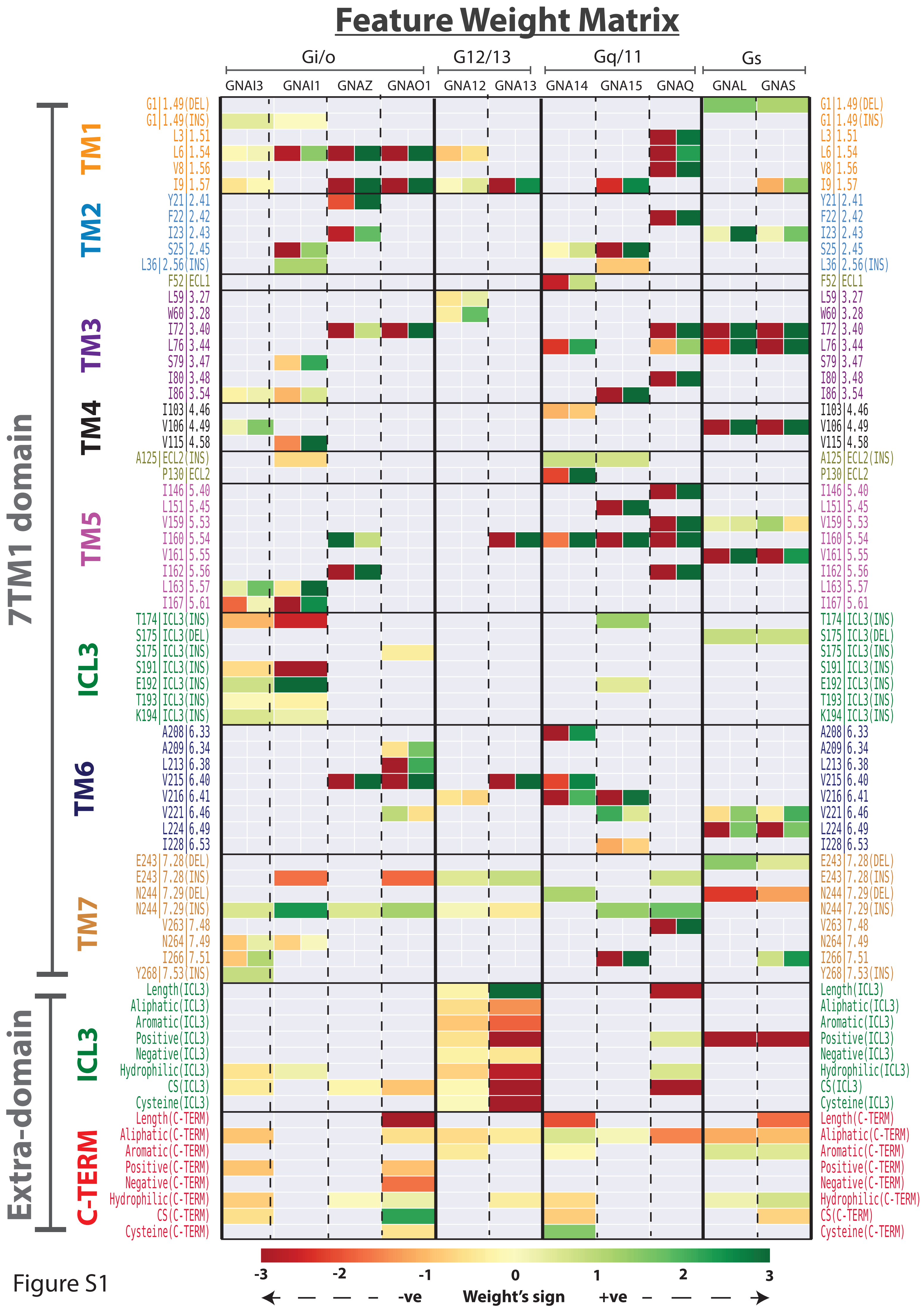

Feature Weight Matrix
Gi/o
G12/13
Gq/11
Gs
GNAI3
GNAI1
GNAZ
GNAO1
GNA12
GNA13
GNA14
GNA15
GNAQ
GNAL
GNAS
7TM1 domain
Extra-domain
TM1
TM2
TM3
TM4
TM5
ICL3
TM6
TM7
ICL3
C-TERM
G1|1.49(DEL)
G1|1.49(INS)
L3|1.51
L6|1.54
V8|1.56
I9|1.57
Y21|2.41
F22|2.42
I23|2.43
S25|2.45
L36|2.56(INS)
F52|ECL1
L59|3.27
W60|3.28
I72|3.40
L76|3.44
S79|3.47
I80|3.48
I86|3.54
I103|4.46
V106|4.49
V115|4.58
A125|ECL2(INS)
P130|ECL2
I146|5.40
L151|5.45
V159|5.53
I160|5.54
V161|5.55
I162|5.56
L163|5.57
I167|5.61
T174|ICL3(INS)
S175|ICL3(DEL)
S175|ICL3(INS)
S191|ICL3(INS)
E192|ICL3(INS)
T193|ICL3(INS)
K194|ICL3(INS)
A208|6.33
A209|6.34
L213|6.38
V215|6.40
V216|6.41
V221|6.46
L224|6.49
I228|6.53
E243|7.28(DEL)
E243|7.28(INS)
N244|7.29(DEL)
N244|7.29(INS)
V263|7.48
N264|7.49
I266|7.51
Y268|7.53(INS)
Length(ICL3)
Aliphatic(ICL3)
Aromatic(ICL3)
Positive(ICL3)
Negative(ICL3)
Hydrophilic(ICL3)
CS(ICL3)
Cysteine(ICL3)
Length(C-TERM)
Aliphatic(C-TERM)
Aromatic(C-TERM)
Positive(C-TERM)
Negative(C-TERM)
Hydrophilic(C-TERM)
CS(C-TERM)
Cysteine(C-TERM)
-3
-2
-1
0
1
2
3
-ve
Weight's sign
+ve

Figure S1

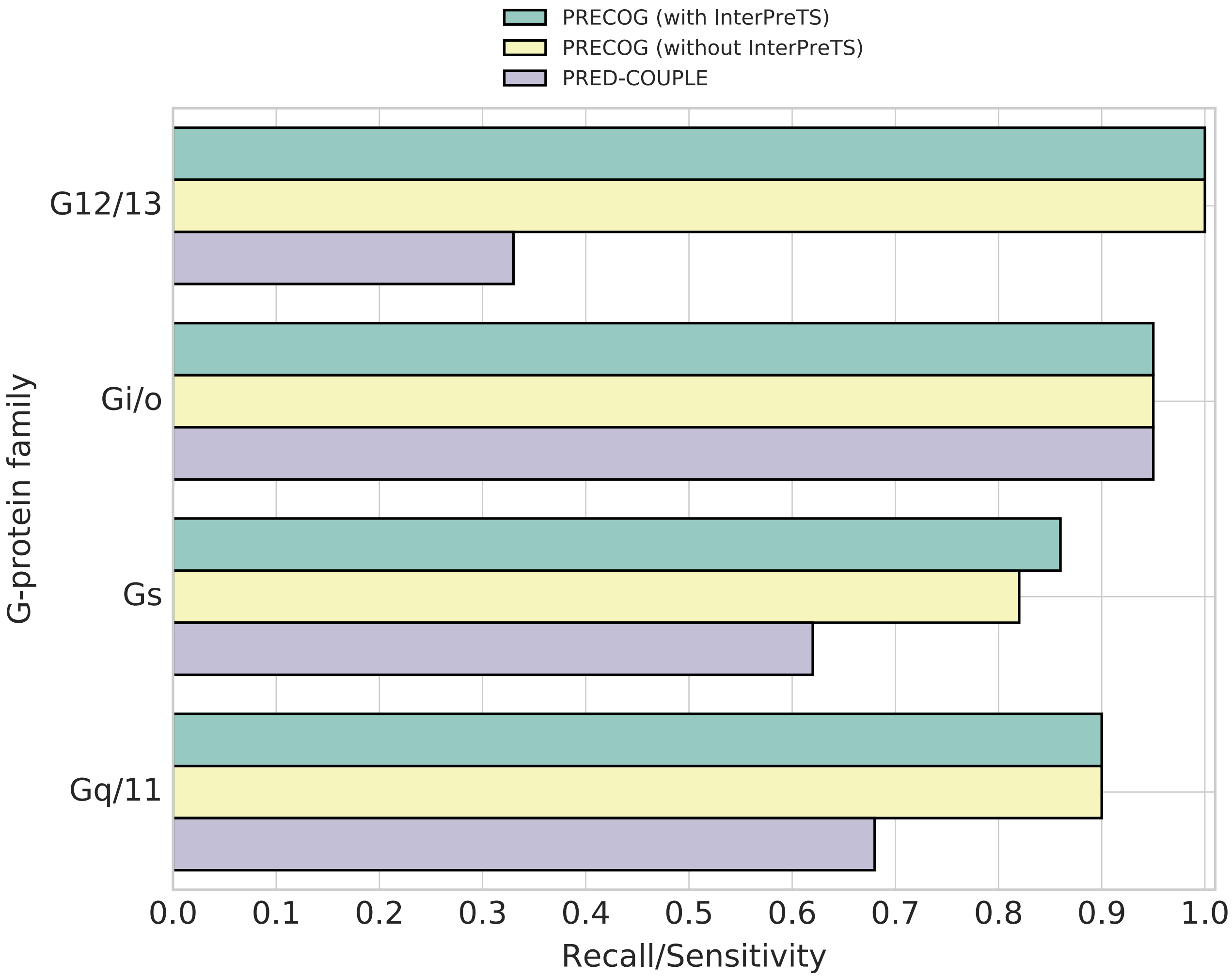

PRECOG (with InterPreTS)
PRECOG (without InterPreTS)
PRED-COUPLE
G12/13
Gi/o
Gs
Gq/11
G-protein family
0.0
0.1
0.2
0.3
0.4
0.5
0.6
0.7
0.8
0.9
1.0
Recall/Sensitivity

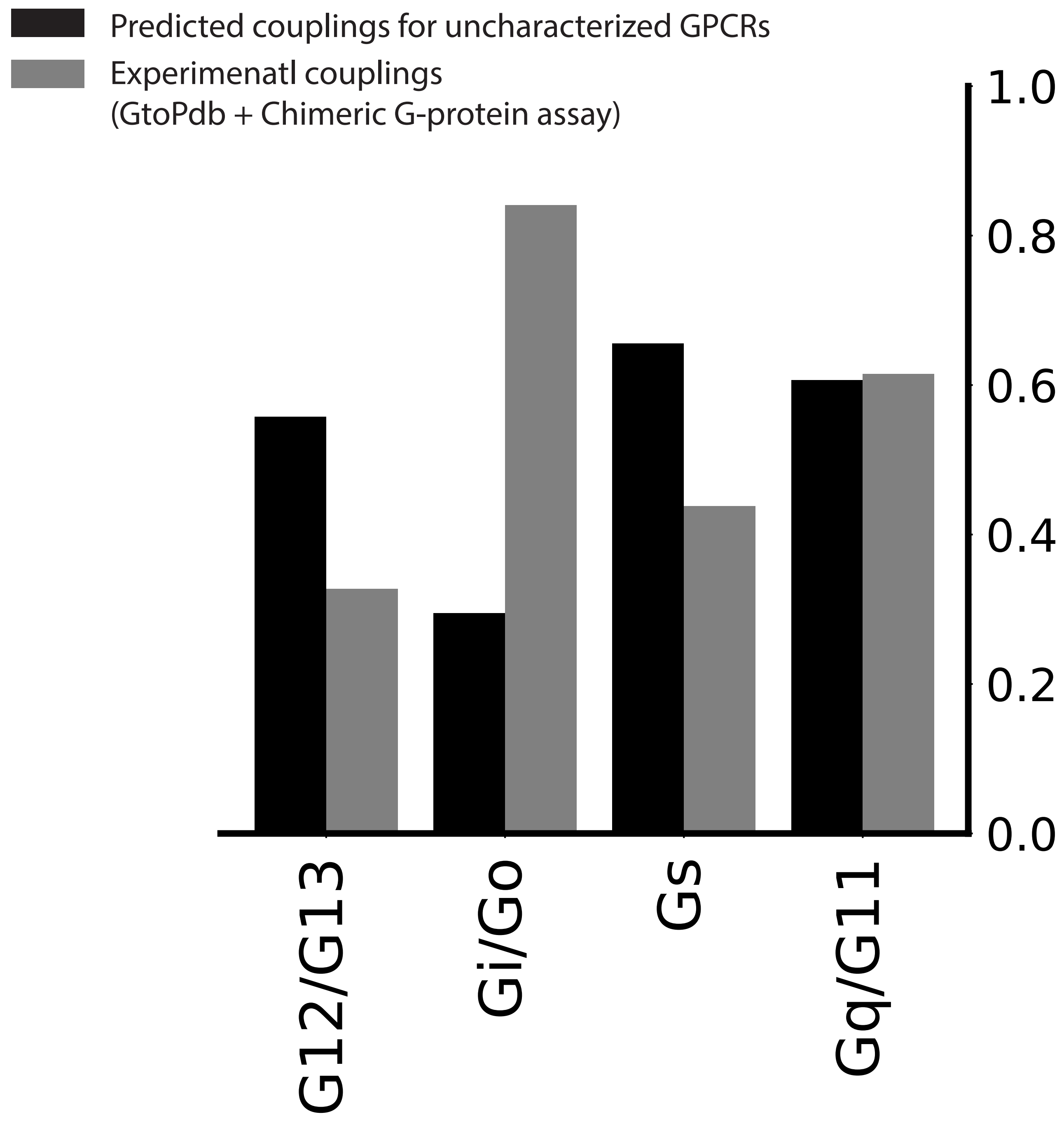
Predicted couplings for uncharacterized GPCRs
Experimenatl couplings
(GtoPdb + Chimeric G-protein assay)
1.0
0.8
0.6
0.4
0.2
0.0
G12/G13
Gi/Go
Gs
Gq/G11
