# Supplementary material for: PRECOG: PREdicting COupling probabilities of G-protein coupled receptors
Source: Nucleic Acids Res. 2019 May 30;47(W1):W395–401. doi: 10.1093/nar/gkz392 (PMC6602504; doi:10.1093/nar/gkz392)

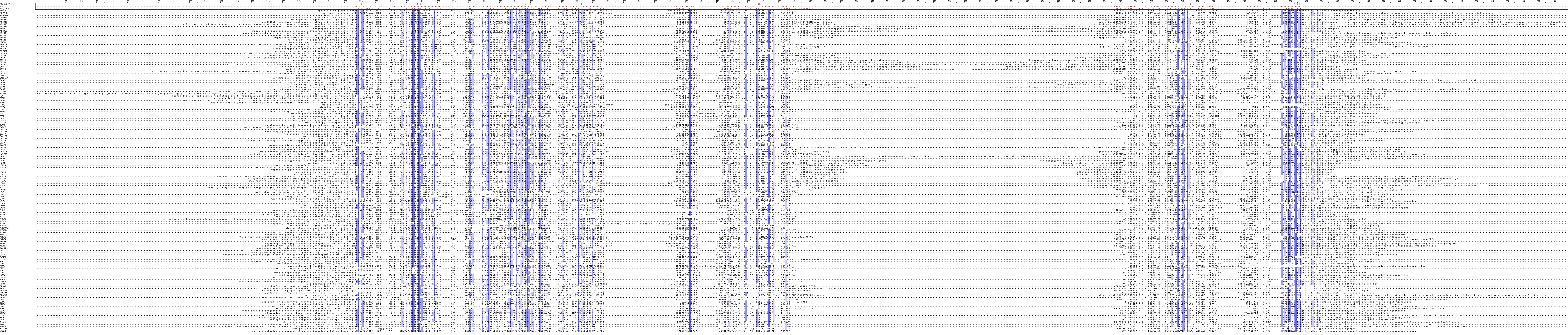

Supplement: gkz392_Supplemental_Files [file Dataset_S2_GPCR_classA_selected_shedding_MSA.pdf › Dataset_S2_GPCR_classA_selected_shedding_MSA.pdf]
